# Clinical and implementation outcomes of an antimicrobial stewardship intervention for rapid blood culture diagnostics

**DOI:** 10.1017/ash.2025.10225

**Published:** 2025-11-26

**Authors:** Monica Abdelnour, Christine R. Lockowitz, Evan E. Facer, Andrew Atkinson, Sara Malone, Virginia R. McKay, Rebekah E. Dumm, Alexander S. Plattner, Matthew M. Sattler, Jason G. Newland

**Affiliations:** 1 Washington University in St. Louis School of Medicine, Department of Pediatrics, Division of Infectious Diseases, St. Louis, MO, USA; 2 Department of Pharmacy, St. Louis Children’s Hospital, St. Louis, MO, USA; 3 Washington University in St. Louis School of Medicine, Division of Infectious Diseases, St. Louis, MO, USA; 4 Institute for Informatics, Data Science and Biostatistics (I2DB), Washington University in St. Louis School of Medicine, St. Louis, MO, USA; 5 School of Public Health, Washington University in St. Louis, St. Louis, MO, USA; 6 Brown School, Washington University in St. Louis, St. Louis, MO, USA; 7 Department of Pathology and Immunology, Washington University in St. Louis School of Medicine, St. Louis, MO, USA; 8 Department of Pediatrics, Division of Infectious Diseases, Nationwide Children’s Hospital and The Ohio State University School of Medicine, Columbus, OH, USA

## Abstract

**Objective:**

To evaluate the clinical and implementation outcomes of an antimicrobial stewardship program (ASP) intervention to improve antibiotic therapy for a rapid diagnostic test (RDT) for bloodstream infections (BSIs).

**Design:**

Retrospective pre and postintervention study.

**Setting:**

Single pediatric tertiary center from August 2022 to May 2024.

**Participants:**

Patients presenting with a positive blood culture accompanied by the Verigene (VG) Gram-positive Blood Culture Nucleic Acid (BC-GP) assay. Implementation outcomes surveys were completed by infectious diseases (ID) clinicians and non-ID clinicians.

**Methods:**

We implemented a 24 hours a day, 7 days a week (24/7) ASP intervention to improve response to BC-GP assay results. The primary clinical end point was time to optimal antimicrobial therapy (OAT). Secondary endpoints included duration of bacteremia, hospital length of stay (LOS), and mortality. We assessed our intervention’s acceptability, appropriateness, and feasibility using validated implementation outcomes surveys.

**Results:**

Among 211 pre and 91 postimplementation BC-GP results, the median time to OAT decreased from 20.3 hours (95% CI:14.2–26.4) to 1.3 hours (95% CI: 0.0–2.7), *p* = .002. No significant differences were found in duration of bacteremia, LOS, or mortality. Implementation surveys from 23 ID and 47 non-ID clinicians demonstrated over 80% agreement on intervention appropriateness and acceptability. ID clinicians rated feasibility measures lower than non-ID clinicians (3.58 vs 4.51 on a five-point Likert scale, *p* < .001).

**Conclusions:**

24/7 ASP intervention paired with RDTs for Gram-positive BSIs is associated with reduced time to OAT. Feasibility differences between ID and non-ID clinicians highlight implementation challenges and the need for future strategy evaluation.

## Introduction

Bloodstream infections (BSIs) in children account for approximately 4% of admissions annually and are associated with a three-fold increased risk of mortality.^1^ BSIs are associated with the highest number of preventable deaths among hospital-acquired infections and the highest costs, ranging from 1–18 billion dollars annually.^2^ Timely antibiotic initiation is associated with reduced morbidity in the setting of sepsis and BSI.^3,4^ However, routine blood culture results require 24–72 hours for pathogen identification and antibiotic susceptibility testing, which delay initiation of optimal antibiotic therapy (OAT).

The Verigene (VG) Gram-positive Blood Culture Nucleic Acid (BC-GP) assay is an FDA-approved rapid diagnostic test (RDT) capable of identifying 9 bacterial species, 3 genera, and 3 resistance markers (*mecA* for *Staphylococcus aureus* or *S. epidermidis,* and *vanA* or *vanB* for *Enterococcus faecalis* or *E. faecium*) within 2.5 hours of Gram stain detection.^5^ Subculturing remains necessary for susceptibility testing and identifying organisms not detected by the test. The BC-GP assay shortens organism identification time, potentially allowing for earlier time to OAT. However, the clinical impact of these RDTs is mixed; some studies show earlier time to OAT, others do not.^6–10^ A potential confounder is the availability of experienced clinicians to assist in the interpretation of RDT results. One study found that ASP clinicians were available to interpret RDT results in 85% of cases during the day, compared to only 15% during the night shift.^16^ While improved time to OAT following the integration of ASP and RDT has been demonstrated in a number of studies,^11–14^ existing literature has not explored the implementation outcomes of feasibility, acceptability, and appropriateness associated with this intervention. Understanding these outcomes is essential for intervention optimization and sustainability across diverse healthcare environments. We aimed to assess the feasibility, acceptability, and appropriateness of an ASP intervention coupled with the BC-GP assay in a tertiary children’s hospital. We also assessed the clinical outcomes including time to OAT, duration of bacteremia, hospital length of stay (LOS), and mortality.

## Methods

### Study design and population

We conducted a retrospective pre and postintervention study (August 2022–May 2024) to assess the impact of an ASP intervention to improve the use of the BC-GP assay result for BSIs. Implementation outcomes were also evaluated. We conducted this study at St. Louis Children’s Hospital (SLCH), a 455-bed academic tertiary children’s hospital with roughly 275,000 patient visits per year. The ASP team at SLCH, composed of an infectious diseases (ID) pharmacist and ID physician, perform prospective audit and feedback via handshake stewardship for hospitalized patients receiving antibiotics during daytime hours Monday through Friday. The BC-GP assay is performed 24 hours a day, 7 days a week (24/7) on the first blood culture, over a 3-day period, with Gram-positive (GP) bacteria identified on Gram stain. Prior to the intervention, the ASP team did not receive real-time BC-GP assay notifications. Antibiotic changes based on BC-GP assay results were recommended only during handshake stewardship rounds (typically 1000–1200).We included patients presenting to SLCH who had a blood culture collected at SLCH with a positive result per the BC-GP assay (BC-GP assay positive blood cultures). A microbiology technician calls the primary clinician with any positive BC-GP assay result. We included only the first BC-GP assay positive blood culture during the index admission. We excluded repeat BC-GP assay positive blood cultures during the index admission. We excluded patients if they had two or more different bacterial species isolated within 48 hours of the initial positive blood culture, or if Gram-negative (GN) bacteria was identified.

### Intervention

The ASP team began 24/7 real-time responses for BC-GP assay results in November 2023 and continued through May 2024 (7 months). During this period, an on-call ASP clinician/trainee was notified 24/7 of BC-GP assay results through an automated alert in the electronic health record (EHR). Overnight EHR notifications were received by the ASP member via an audible alert, and patient charts were reviewed via mobile EHR application. ASP team members shared on-call responsibilities on a rotating schedule; team members consisted of three pediatric ID fellows, two pediatric ID physicians, and one ID pharmacist. Upon alert, the ASP member reviewed the BC-GP assay result, and all relevant patient specific factors (eg, immune status, central venous catheters). The ASP member then contacted the appropriate medical or surgical clinician by telephone with recommendations and documented these recommendations in the EHR, including time of contact with the clinician.

We created a guidance document to standardize antibiotic recommendations and dosing based on assay result and illness severity, prior to the intervention (Supplemental table 1 and 2). If the patient was already receiving OAT, based on assessment by the ASP team, no intervention was provided. Prior to the start of the intervention, we presented an in-person education session to impacted clinical services. Sessions covered the literature supporting the BC-GP assay with ASP intervention, the local algorithm, and the logistics of the 24/7 ASP intervention. Clinical services had the opportunity to specify their preferred contact for the ASP recommendations (eg, trainee, nurse practitioner, or attending).

### Outcomes

The primary clinical outcome was time (in hours) to OAT, calculated as time from BC-GP assay result to time the order of the optimal antibiotic agent(s) was placed in the EHR. We defined OAT as the most narrow-spectrum and effective antibiotic regimen for the identified organism and clinical syndrome, agreed upon by the ASP team as outlined in the guidance document (Supplemental table 1 and 2). If the ASP team member deemed the detected organism a contaminant, OAT was defined as discontinuation of empiric antibiotics not otherwise indicated for the treatment of a different infection. If the patient was receiving OAT at the time of the BC-GP assay result, the time of OAT initiation was calculated and recorded as a negative value. Secondary clinical outcomes included (1) duration of bacteremia, defined as days of positive blood culture(s), (2) hospital LOS, measured in days from admission to discharge times, and (3) mortality defined as the occurrence of death from any cause during each study period.

We assessed the acceptability, appropriateness, and feasibility of the ASP intervention using validated surveys to two groups of clinicians to identify key factors associated with its implementation.^15^ Preintervention, we assessed the ID clinicians’ perceptions of implementing this 24/7 intervention. We distributed this survey to 28 members of the ID division to evaluate the baseline perception of our intervention. ID clinicians included attendings, fellows, nurse practitioners, and pharmacists. The ASP team conducting the intervention were members of the ID division and not differentiated from other ID clinicians due to the anonymous nature of the survey. During the intervention we assessed the same perceptions of frontline clinicians within 24-hours of receiving the ASP recommendation. Frontline clinicians included attendings, fellows, residents, and nurse practitioners across SLCH. These surveys used a 5-point Likert scale from strongly disagree (1) to strongly agree (5). We estimated the time required to complete real-time ASP interventions as an objective assessment of feasibility, calculated as the time from the BC-GP assay result to the time of ASP contact to the frontline clinician.

### Data collection and statistical analysis

We collected patient demographics (eg, age, sex, race, and ethnicity) and clinical characteristics (eg, antibiotic allergy, immune status, and gestational age) retrospectively via EHR review. We collected preintervention data from August 2022 to September 2023 from patients with the first BC-GP assay positive blood cultures.

We compared categorical data using chi-squared or Fisher exact tests for smaller sample sizes. Clinical outcomes are presented as medians with an associated interquartile range (IQR). We assessed time to OAT (overall and in three 8-hour shifts, 0000–0800, 0800–1600, and 1600–0000), duration of bacteremia and hospital LOS using the Mann–Whitney U test. We used chi-squared statistical analysis to assess the clinical outcome of mortality. Kaplan-Meier (KM) survival analysis with log rank tests compared overall time to OAT between study periods. We analyzed implementation survey results using independent samples t-tests. We compared the estimated time requirement for real-time ASP interventions across shifts using the Kruskal-Wallis H test. We considered a *p*-value less than .05 statistically significant. We performed all statistical analysis using IBM SPSS statistics version 29.0.2.0 (20).

This study was exempt from full Institutional Review Board (IRB) approval as it was classified as quality improvement.

## Results

### Patient cohort and demographics

**Figure 1.**
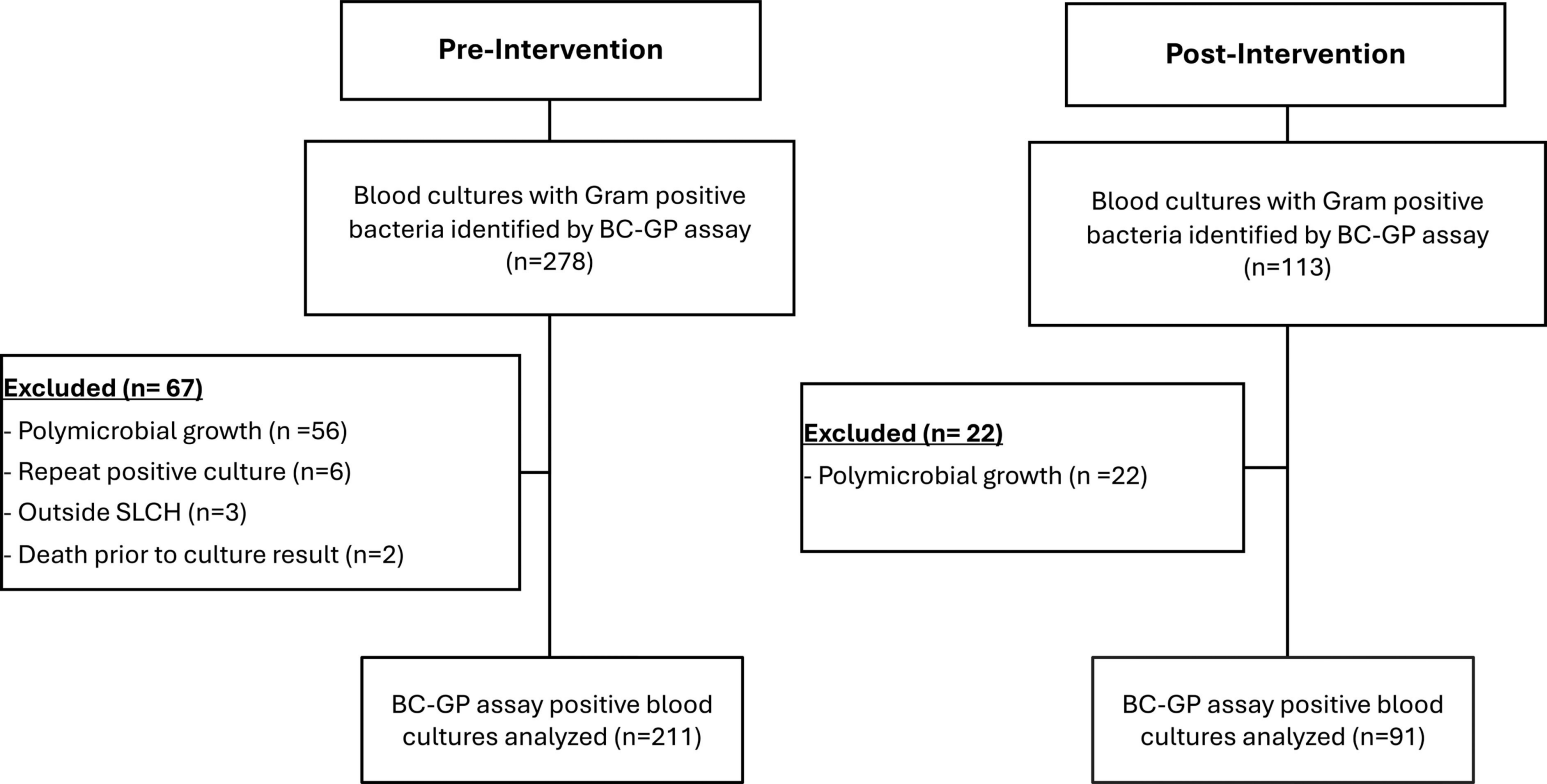
BC-GP assay positive blood culture distribution in the pre and postintervention groups. Abbreviations: BC-GP, gram-positive blood culture, SLCH, St. Louis Children’s hospital.

In the preintervention period, 278 BC-GP assay positive blood cultures were identified. After excluding 67 cultures (56—polymicrobial growth, 2—finalized after patient death, 3—not obtained at SLCH, and 6—repeat positive), a total of 211 individual BC-GP assay positive blood cultures remained for analysis. Of the 113 BC-GP assay positive blood cultures in the postintervention period, we excluded 22 for polymicrobial growth, resulting in a total of 91 BC-GP assay positive blood cultures (Figure 1). The median (IQR) age in the preintervention period was 2.5 years (.1, 10.5) and consisted of 122 (57%) males. Children in the preintervention period were predominantly white (139, 67%) and non-Hispanic (197, 93%). Other than immunocompromised status, demographics of children in the postintervention period did not differ significantly from those in the preintervention period (Table 1). Methicillin-resistant *Staphylococcus epidermidis* was the most commonly identified organism by the BC-GP assay in both groups. However, there was a significant difference in organism detection on the BC-GP assay between the two groups, primarily in the number of methicillin-susceptible *Staphylococcus epidermidis* isolates (Table 1).

**Table 1.** Demographics of children with positive BC-GP assays

	Preintervention (N = 211)	Postintervention (N*=*91)	*p*- value
Age (years), median (IQR)	2.5 (.1, 10.5)	2.3 (.1, 12.6)	.760^a^
Sex, male, *n* (%)	121 (57.3)	51 (56.0)	.834
Race, *n* (%)			.641
White	139 (65.9)	68 (74.7)	
Black	53 (25.1)	17 (18.7)	
Asian	5 (2.4)	1 (1.1)	
Mixed	6 (2.8)	2 (2.2)	
Other	8 (3.8)	3 (3.3)	
Ethnicity, *n* (%)			.599
Hispanic	10 (4.7)	6 (6.6)	
Non-Hispanic	197 (93.4)	82 (90.1)	
Unknown	4 (1.9)	3 (3.3)	
Antibiotic allergy, *n* (%)	48 (22.7)	16 (17.6)	.313
Immunocompromised, *n* (%)	59 (28.0)	14 (15.4)	.019
Premature, *n* (%)	38 (18.0)	22 (24.2)	.218
Neutropenic, *n* (%)	17 (8.1)	4 (4.4)	.185^b^
ICU Status, *n* (%)	98 (46.4)	38 (41.8)	.453
Organism detected on BC-GP assay, *n* (%)			.008
Methicillin-susceptible *Staphylococcus aureus*	30 (14.2)	18 (19.8)	
Methicillin-resistant *Staphylococcus aureus*	10 (4.7)	6 (6.6)	
Methicillin-resistant *Staphylococcus epidermidis*	49 (23.2)	27 (29.7)	
Methicillin-susceptible *Staphylococcus epidermidis*	21 (10.0)	1 (1.1)	
*Staphylococcus* species	35 (16.6)	6 (6.6)	
*Staphylococcus lugdunensis*	2 (.9)	0 (.0)	
*Streptococcus agalactiae*	5 (2.4)	6 (6.6)	
*Streptococcus pneumoniae*	4 (1.9)	5 (5.5)	
*Streptococcus pyogenes*	11 (5.2)	3 (3.3)	
*Streptococcus anginosus* group	4 (1.9)	1 (1.1)	
* Streptococcus* species	21 (10.0)	15 (16.5)	
Vancomycin-susceptible *Enterococcus faecalis*	18 (8.5)	3 (3.3)	
Vancomycin-resistant *Enterococcus faecium*	1 (.5)	0 (.0)	
Blood culture contamination, *n* (%)	82 (38.9)	34 (37.4)	.806
Infectious diseases consult, *n* (%)	150 (71.1)	68 (74.7)	.518

Abbreviations: IQR, Interquartile range, ICU, Intensive Care Unit

a *p* values reported using Mann–Whitney U statistical analysis. Significant value: *p*< .05

During the postintervention period, frontline clinicians ordered 194 antibiotics for 91 BC-GP assay positive blood cultures, on which the ASP team intervened on 125 (64%; see Supplemental table 3). The ASP team recommended discontinuation of antibiotics in over half of the interventions (72/125, 58%). Vancomycin was the most frequently recommended antibiotic to discontinue (27/72, 38%). Frontline clinicians accepted ASP recommendations in 94% of cases (117/125) and acceptance did not significantly differ based on the type of ASP recommendation (continuation, initiation, or discontinuation; see Supplemental table 4).

### Clinical outcomes

**Figure 2.**
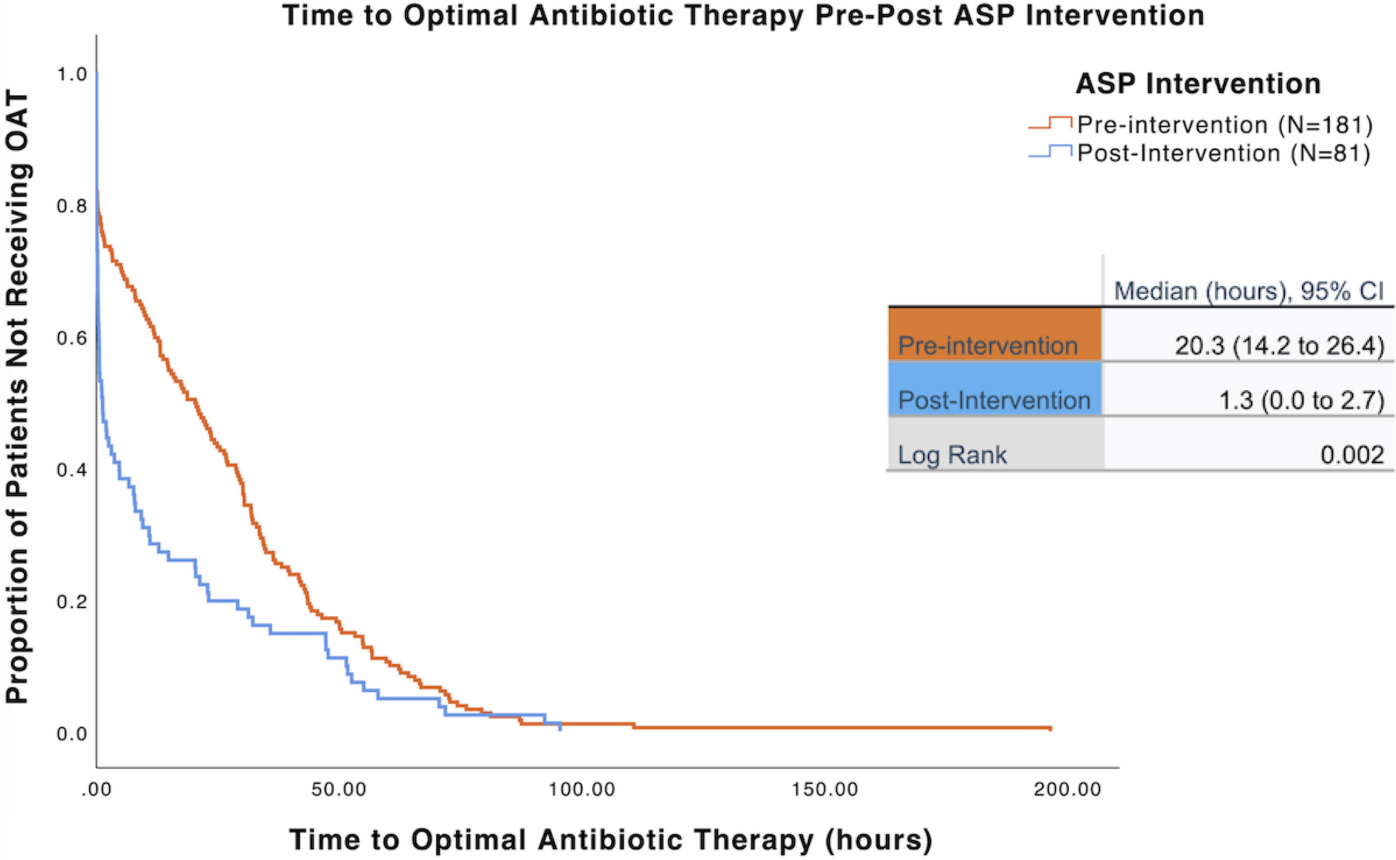
Kaplan-Meier estimates for proportion of patients not receiving OAT, stratified by intervention. Figure 2 demonstrates the Kaplan-Meier survival curve comparing the proportion of patients not receiving OAT over time (hours) in the preintervention (orange) and postintervention (blue) groups. The postintervention group has a smaller proportion of patient not receiving OAT, with a shorter time (hours) to OAT, compared to the preintervention group. Median times to OAT (95% CI) illustrate this difference. Log rank analysis was used to compare the median times, with a statistically significant defined as < .05. Abbreviations: OAT, optimal antibiotic therapy, CI, confidence interval, ASP, antimicrobial stewardship program.

**Figure 3.**
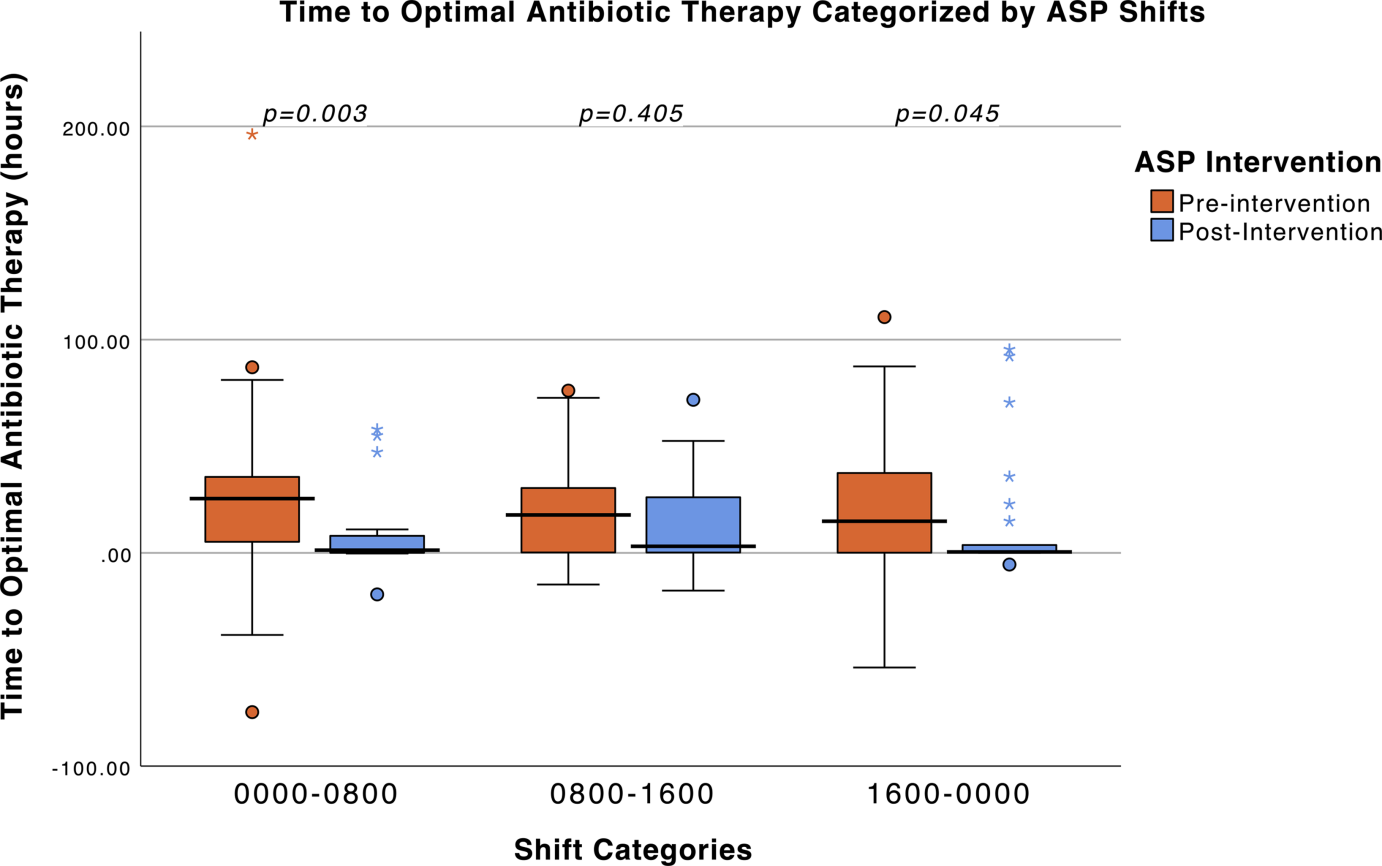
Boxplots of time to OAT in patients pre-post ASP intervention, stratified by shift. Box plot comparing time to OAT stratified by three shifts in the pre and postintervention groups. The IQR is represented by boxes, with the horizontal line inside the box indicating the median time to OAT (hours). Box whiskers extend to the maximum and minimum values within 1.5 times the IQR. Mild outliers, outside 1.5 times the IQR, are marked with a circle, and extreme outliers, outside 3 times the IQR, are marked with an asterisk. Statistical differences between groups are assessed using Mann–Whitney U, and significant values are defined at *P* < .05. Shift 0000–0800 preintervention (*n* = 64), postintervention (*n* = 25). Shift 0800–1600 preintervention (*n* = 66), postintervention (*n* = 35). Shift three 1600–0800 preintervention (*n* = 63), postintervention (*n* = 26). Abbreviations: OAT, optimal antibiotic therapy, ASP, antimicrobial stewardship program.

We observed a significant reduction in overall time to OAT, from 20.3 hours (95% CI:14.2–26.4) in our preimplementation period to 1.3 hours (95% CI: 0.0–2.7) post 24/7 ASP intervention by KM survival analysis (Figure 2). The log rank test revealed a significant difference between the pre and postintervention time to OAT (*p* = .002). Stratifying by shift, we observed a significant difference during the 0000–0800 shift, with a decrease in median time (IQR) to OAT from 25.5 hours (5.1, 36.0) in the preintervention period to 1.3 hours (0.0, 8.8) postintervention, *p* = .003 (Figure 3). We also observed a significant difference during the 1600–0000 shift, with a decrease in median time to OAT from 14.8 hours (0.0, 38.1) in the preintervention period to 0.5 hours (0.1, 6.5) postintervention (*p* = .045). During the hours of 0800–1600, there was a decrease in median time to OAT from 17.8 hours (0.2, 31.4) to 3.1 hours (0.1, 29.1) in the pre and postintervention periods, respectively, however this difference was not statistically significant (*p* = .405). There were no statistically significant differences in hospital LOS, duration of bacteremia, or mortality between the pre and postintervention periods (Table 2).

**Table 2.** Secondary clinical outcomes pre-post ASP intervention

	Preintervention	Postintervention	*p*-value
Duration of bacteremia (days), median (IQR)	N = 128 2.0 (1.0, 3.0)	N = 56 1 (1.0, 3.0)	.245^a^
Hospital length of stay (days), median (IQR)	N = 202 13.1 (4.8, 57.4)	N = 89 15.0 (5.7, 47.1)	.808^a^
In-hospital mortality, *n* (%)	N = 211 19 (.1)	N = 91 9 (.1)	.808

Abbreviations: ASP, Antimicrobial Stewardship Program, IQR, Interquartile Range

a *p* values reported using Mann–Whitney U statistical analysis Significant value: *p*< 0.05.

### Implementation outcomes

**Figure 4.**
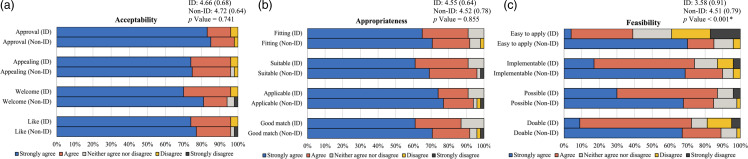
Implementation outcomes of appropriateness, acceptability, and feasibility grouped by ID and non-ID clinicians. (a) displays the survey results for the implementation outcome of appropriateness in ID compared to non-ID clinicians. (b) displays the survey results for the implementation outcome of acceptability in ID compared to non-ID clinicians. (c) displays the survey results for the implementation outcome of feasibility in ID compared to non-ID clinicians. The overall mean (SD) for the 4 questions in each domain is compared using independent samples t-tests, and significant values are defined at *p*< .05 and indicated by asterisks (*). Abbreviations: ID, infectious diseases, SD, standard deviation.

Twenty-three ID clinicians completed the preimplementation survey (82% response rate). The majority of respondents, 70%, were attending physicians. Forty-seven non-ID clinicians completed the survey during the ASP intervention (55% response rate). The majority, 40%, of respondents were resident physicians. The average (standard deviation) acceptance of the intervention, evaluated on a 5-point Likert scale, did not differ significantly between ID and non-ID clinicians (4.66 [0.68] vs 4.72 [0.64], *p* = .741). Similar results were seen for appropriateness (4.55 [0.68] vs 4.55 [0.64], *p* = .855). However, we observed significantly lower average scores by ID clinicians compared to non-ID clinicians in the feasibility domain (3.58 [0.91] vs 4.51 [0.79], *p*< .001). Specifically, ID clinicians’ perception of the intervention’s “implementability,” “doability,” and “ease of application” differed significantly from non-ID clinicians. The largest difference was in the “ease of application” responses, with lower average scores by ID clinicians compared to non-ID clinicians (2.87 [1.22] vs 4.50 [0.86], *p*< .001). Responses to the surveys are summarized in Figure 4.

During the postimplementation period, the ASP team received 113 BC-GP assay notifications, 70 (62%) of which required intervention to achieve OAT. We excluded two interventions from time calculations as the ASP intervention occurred before BC-GP assay results were provided to frontline clinicians. The remaining 68 ASP interventions had an estimated median time requirement of 10.5 minutes (6.0, 19.0). There was no statistically significant difference in time requirement between the ASP shifts, *p* = .438 (Table 3). We did not calculate time estimates for the 43 BC-GP assay results that did not require ASP intervention.

**Table 3.** Time requirement for ASP interventions per shift

		**Total**	**00:00–08:00**	**08:00–16:00**	**16:00–00:00**	
**ASP intervention**	Yes, *n* (%)	68 (61.3)	18 (54.5)	24 (58.5)	26 (70.3)	*p*-value
	Estimated time (minutes), Median (IQR)	10.5 (6.0, 19.0)	8.5 (4.0, 16.3)	15.0 (31.5, 6.0)	9.5 (23.5, 5.8)	.438^*^
**Total BC-GP Assay Results, *n***		111	33	41	37	

Abbreviations: ASP, Antimicrobial Stewardship Program, BC-GP, Gram-Positive Blood Culture Nucleic Acid

* *p*-value reported using Kruskal-Wallis H statistical analysis. Significant value: *p*< .05.

## Discussion

This study demonstrates the ability to decrease time to OAT through the integration of a 24/7 real-time ASP intervention coupled with RDT results for BSIs in children. Discontinuation of antibiotics, particularly vancomycin, was the most common intervention required to achieve OAT. Frontline clinicians accepted ASP recommendations equally for antibiotic continuation, initiation, and discontinuation. These findings align with Tribble *et al*.’s study,^16^ which demonstrated improved antibiotic management in children with ASP interventions from 0700 to 2 300 Monday through Sunday paired with RDTs. Previous literature has noted the impact of day versus night shift on the antibiotic management.^17^ Therefore, we compared the time to OAT at different times of day. Our study showed a significant reduction in time to OAT during the evening and night shifts (1600–0000 and 0000–0800). Interestingly, we did not observe a significant difference in time to OAT during day shift (0800–1600). Data review demonstrated that the lack of day-shift improvement was due to consistent delays in time to OAT, not outliers. Contributing factors may include resident floor responsibilities, increased house staff presence and antibiotic discussions, and resident daytime meetings. This underscores the importance of stakeholder engagement across all levels of the healthcare hierarchy to ensure consistent intervention implementation. Although not significant, the ASP intervention time increased slightly during day-shift; possibly contributing to the observed delay in time to OAT.

A recently published meta-analysis and systematic review, including 12 pediatric studies, demonstrated a decrease in time to OAT, mortality, and LOS.^18^ However, many of the studies included rapid blood culture diagnostics with the addition of GN organism identification, which may be associated with increased mortality in the absence of OAT.^19^

Our study evaluated the implementation outcomes for the integration of ASP intervention in parallel with RDTs. We chose to use validated quantitative surveys^15^ to assess the three implementation outcomes, as the shorter format encouraged completion. The implementation survey revealed the ID clinicians’ hesitancy about the intervention’s feasibility, with specific concerns regarding its ease of application and implementable nature. This is likely due to the time constraints in the ID division and a lack of incentives for project initiation. However, non-ID clinicians reported strong acceptance, appropriateness, and feasibility in association with improved clinical outcomes of time to OAT. This heightened the interest for continuing the 24/7 intervention and has led the SLCH ID division, as a whole, to implement ongoing BC-GP assay result monitoring and intervention. Maintenance of the intervention from 1600–0800 Monday through Friday and weekends will continue, provided by the ID clinician on-call (fellow or faculty). An ASP team member will maintain the intervention Monday through Friday, 0800–1600. Despite the ID division’s growing efforts, feasibility concerns noted by the implementation outcome results may affect sustainability. Some studies have highlighted the role of electronic decision support systems in improving the use of RDTs.^20^ Despite promising results, sustainability has not been fully assessed, and foundational education on OAT remains essential. For long term success and sustainability, a potential strategy is to secure additional funding through a cost analysis evaluating the return on investment from saved antibiotics per patient day.

Based on the ID clinicians’ feasibility concerns, we estimated the time required for real-time ASP interventions. This calculation is likely an underrepresentation, as it does not account for the time spent on telephone conversations between the frontline clinician and the ASP team member, nor the additional time required for documentation of each intervention. Time required to review BC-GP assay results, when the patient was on OAT at the time of the result, was not calculated as no documentation was completed in these cases. Additionally, stakeholder education and engagement were integral to the success of our intervention. Therefore, dedicating time to prepare and present our planned intervention to impacted clinical services was essential prior to the intervention period. Finally, the RDT used at SLCH during our study detected only GP organisms, so time requirements may be higher in institutions using panels that include GN and/or fungal targets.

Limitations of this study included its single center design, limiting reproducibility across institutions with similar RDTs but differing stewardship support. Observation bias by the primary team may have exaggerated the reduction in time to OAT in the postintervention period. We did not identify a significant decrease in duration of bacteremia, hospital LOS, or mortality, likely as most interventions aimed to discontinue antibiotics. Additionally, Staphylococcus epidermidis was the most common organism identified. Given its higher likelihood of being a contaminant, this raises concern that a substantial proportion of contaminated blood cultures may have diluted the observed impact of the ASP intervention on clinical outcomes. Demographic variables were generally well balanced between groups, except for a statistically significant difference in the proportion of immunocompromised patients. This imbalance may have affected clinical outcomes; however, the study was not powered for subgroup analysis. Ideally, ASP members would have been surveyed separately from other ID clinicians. However, due to concerns about maintaining anonymity with only six ASP members, the decision was made to survey all ID clinicians together. Finally, quantitative surveys were utilized to assess our implementation outcomes, as they were efficient for clinicians to complete. However, the exclusion of qualitative surveys limited the identification of specific barriers and facilitators which may be addressed in future work.

In conclusion, 24/7 ASP intervention paired with RDTs is associated with reduced time to OAT and is deemed appropriate and acceptable by ID and non-ID clinicians. However, the difference in feasibility perception by ID and non-ID clinicians highlights implementation challenges, and warrants evaluation of future strategies.

## Supporting information

10.1017/ash.2025.10225.sm001 Abdelnour et al. supplementary material Abdelnour et al. supplementary material

## Supplementary material

The supplementary material for this article can be found at https://doi.org/10.1017/ash.2025.10225
