# Supplementary material for: Clinical and implementation outcomes of an antimicrobial stewardship intervention for rapid blood culture diagnostics
Source: Antimicrob Steward Healthc Epidemiol. 2025 Nov 26;5(1):e313. doi: 10.1017/ash.2025.10225 (PMC12722554; doi:10.1017/ash.2025.10225)
Supplement: Abdelnour et al. supplementary material [file S2732494X25102258sup001.pdf]

Supplementary Tables

Table 1. Recommended Antibiotic Therapy based on Verigene Result and Clinical Scenario		
**Organism**	**Preferred Empiric therapy**	**Alternative Empiric Therapy & Additional considerations**
Enterococcus faecalis[1]	Ampicillin	Vancomycin
Enterococcus faecium (van A and van B negative)	Vancomycin	Linezolid OR Daptomycin[2]
Enterococcus faecium (van A or van B positive, aka VRE)	Linezolid	Daptomycin
Listeria species	Ampicillin	Trimethoprim-sulfamethoxazole
Staphylococcus aureus, mecA negative (MSSA)	Nafcillin/Oxacillin	Cefazolin
Staphylococcus aureus, mecA positive (MRSA)	Ceftaroline OR Daptomycin	Vancomycin
Staphylococcus epidermidis[3]*,* mecA negative (MSSE)	See MSSA section	See MSSA section
Staphylococcus epidermidis[3]*,* mecA positive (MRSE)	Vancomycin	Daptomycin OR Ceftaroline<br>• Repeat blood culture if concern for infection (prior to initiation of targeted antibiotics, if possible)
Staphylococcus lugdunensis	Vancomycin	Daptomycin OR Ceftaroline<br>• Repeat blood culture if concern for infection (prior to initiation of targeted antibiotics, if possible)
Staphylococcus species[3]	Vancomycin	Daptomycin OR Ceftaroline<br>• Repeat blood culture if concern for infection (prior to initiation of targeted antibiotics, if possible)
Streptococcus agalactiae (group B streptococcus, GBS)	Penicillin G OR Ampicillin	Non-severe penicillin allergy:<br>• Consider penicillin allergy de-labeling OR ceftriaxone<br>Severe penicillin allergy[4]:<br>• Vancomycin
Streptococcus anginosus group[5]	Penicillin G OR Ampicillin	Vancomycin
Streptococcus pneumoniae	<u>Meningitis/CNS infection:</u><br>Ceftriaxone and Vancomycin<br><u>Critical Illness[6]:</u><br>Ceftriaxone<br><u>Bacteremia:</u><br>Penicillin G or Ampicillin	Vancomycin
Streptococcus pyogenes	Penicillin G or Ampicillin	Non-severe penicillin allergy:<br>• Consider penicillin allergy de-labeling OR Cefazolin<br>Severe penicillin allergy:<br>• Vancomycin

Streptococcus species[7]	Hematologic malignancy, HSCT patient or Meningitis/CNS infection:<br>Ceftriaxone and Vancomycin<br>Other patients:<br>Penicillin G OR Ampicillin	Vancomycin

Abbreviations: CNS, Central Nervous System, HSCT, Hematopoietic Stem Cell Transplant

1. In the setting of polymicrobial/intra-abdominal infection, consider piperacillin-tazobactam
2. Consider daptomycin in patients on ECMO. Avoid Daptomycin in the setting of respiratory infections.
3. Consider contamination unless clinically appropriate. Organism identification in the setting of hardware, may indicate infection in the appropriate clinical setting.
4. Severe penicillin allergy symptoms include hives, angioedema, respiratory distress, hypotension, anaphylaxis. Other considerations to avoid penicillin include history of Steven-Johnson syndrome (SJS), serum sickness, acute interstitial nephritis, hemolytic anemia or drug rash with eosinophilia and systemic symptoms (DRESS) with penicillin use.
5. Consider empiric therapy with ceftriaxone and metronidazole if there is concern for sinus disease with intracranial extension.
6. Respiratory support with positive pressure ventilation, mechanical ventilation or oscillatory ventilation, hemodynamic support with 1 or more vasoactive medications, severe organ dysfunction (ECMO requirement, coagulopathy, renal replacement therapy) or alterations in mental status.
7. May still be *Streptococcus pneumoniae* given the Verigene systems difficulty with differentiating *Streptococcus pneumoniae* from *Streptococcus mitis/oralis*

Table 2. Unique Dosing Strategies for Certain Antibiotics based on Clinical Scenario		
**Antibiotic**	**Dosing**	**Clinical Pearls**
Daptomycin (assuming normal renal function)	**Neonates with all organisms:**<br>10 mg/kg/dose q12h<br><br>**Non-neonates with MSSA/MRSA based on Package Insert Dosing (see caveats and consider on case by case basis):**<br>≤ 6 years: 12 mg/kg/dose q24h<br>7 to ≤11 years: 9 mg/kg/dose q24h<br>12 to ≤17 years: 7 mg/kg/dose q24h<br>≥18 years: 6 mg/kg/dose q24h (I would do at least 8 mg/kg/dose in this age group and treat with adult dosing)<br><br>**Non-neonates with *Enterococcus faecium:***<br>10-12 mg/kg/dose q24h	• Dose based on adjusted body weight if actual body weight is >120% ideal body weight. Technically no max dose.<br>• Baseline and weekly CK monitoring.<br>• For MSSA/MRSA bacteremia in non-neonates, reasonable to use 8-10 mg/kg/dose in most infections as available PK/PD data suggests current dosages may not achieve adequate target attainment. Clinical/safety data for higher doses are not available in children.<br>• Frequency decrease to q48h if CrCl <30 ml/min or receiving IHD. Dose after dialysis session on dialysis days.
Ceftaroline (assuming normal renal function)	**Neonates:** 6 mg/kg/dose q8h<br><br>**Infants and Children:** 15 mg/kg/dose q8h (max 600 mg/dose)	Requires renal dose adjustment
Nafcillin/Oxacillin	**Non-neonates:** 200 mg/kg/day (max 12g/day) divided q4-6 hours. Consider q4h for bacteremia if feasible.	Increased target attainment given time-dependent drugs.
Ceftriaxone	**Critically ill with hemodynamic instability and hypoalbuminemia:**<br>Reasonable to recommend 50 mg/kg/dose (max 2,000 mg/dose) q12h (instead of typical q24h)	• Highly protein bound with significantly increased clearance of this drug in setting of sepsis and hypoalbuminemia. This increases percent target attainment with minimal risk.<br>• If neonate, ensure age/labs appropriate before using this drug.<br>• If neonate, check if receiving IV calcium or TPN.

Abbreviations: MSSA, Methicillin-susceptible *Staphylococcus aureus,* MRSA, Methicillin-resistant *Staphylococcus aureus*, CK, Creatinine Kinase, CrCl, Creatinine Clearance, PK, Pharmacokinetics, PD, Pharmacodynamics, IHD, Intermittent Hemodialysis, IV, Intravenous, TPN, Total Peripheral Nutrition

Table 3: Post-Intervention Antibiotic Frequency and ASP Interventions						
	Frequency	ASP Intervention N (%)	Continue	Discontinue	Initiate	No ASP intervention
Amoxicillin	1	0 (0%)	0	0	0	1
Ampicillin	16	13 (81%)	1	3	9	3
Amp/sulbactam	1	1 (100%)	0	1	0	0
Cefazolin	18	14 (78%)	0	4	10	4
Cefepime	30	15 (50%)	1	14	0	15
Ceftaroline	5	3 (60%)	0	0	3	2
Ceftazidime	3	2 (67%)	1	1	0	1
Ceftriaxone	28	19 (68%)	2	14	3	9
Ciprofloxacin	1	0 (0%)	0	0	0	1
Clindamycin	3	2 (67%)	0	2	0	1
Daptomycin	2	1 (50%)	0	0	1	1
Gentamicin	4	3 (75%)	0	3	0	1
Meropenem	2	0 (0%)	0	0	0	2
Metronidazole	5	2 (40%)	1	1	0	3
Nafcillin	3	2 (67%)	0	0	2	1
Oxacillin	3	2 (67%)	0	2	0	1
Piperacillin/tazobactam	1	1 (100%)	0	0	1	0
Vancomycin	58	39 (67%)	9	27	3	19
Penicillin G	4	3 (75%)	0	0	3	1
No Antibiotic	6	3 (50%)	3	0	0	3
Total	194	125 (64%)	18	72	35	69

Abbreviations: ASP, Antimicrobial Stewardship Program

Table 4: Acceptance of ASP intervention Grouped by Type of Recommendation			
	Intervention Acceptance		
	Yes	No	Total
Continue (N, %)	18 (100)	0 (0)	18
Discontinue (N, %)	65 (90)	7 (10)	72
Initiate (N, %)	34 (97)	1 (3)	35
**Total (N, %)**	**117 (94)**	**8 (6)**	**125**
**Chi-Square Significance value (*p*-value)**			**0.193**

Abbreviations: ASP, Antimicrobial Stewardship Program
